# Elevated Levels of Cytokines Associated with Th2 and Th17 Cells in Vitreous Fluid of Proliferative Diabetic Retinopathy Patients

**DOI:** 10.1371/journal.pone.0137358

**Published:** 2015-09-09

**Authors:** Masaru Takeuchi, Tomohito Sato, Atsushi Tanaka, Tadashi Muraoka, Manzo Taguchi, Yutaka Sakurai, Yoko Karasawa, Masataka Ito

**Affiliations:** 1 Department of Ophthalmology, National Defense Medical College, Saitama, Japan; 2 Department of Developmental Anatomy and Regenerative Biology, National Defense Medical College, Saitama, Japan; Oregon Health & Science University, UNITED STATES

## Abstract

Macrophages are involved in low-grade inflammation in diabetes, and play pathogenic roles in proliferative diabetic retinopathy (PDR) by producing proinflammatory cytokines. T cells as well as other cells are also activated by proinflammatory cytokines, and infiltration into the vitreous of patients with PDR has been shown. In this study, we measured helper T (Th) cell-related cytokines in the vitreous of PDR patients to define the characteristics of Th-mediated immune responses associated with PDR. The study group consisted of 25 type 2 diabetic patients (25 eyes) with PDR. The control group consisted of 27 patients with epiretinal membrane (ERM), 26 patients with idiopathic macular hole (MH), and 26 patients with uveitis associated with sarcoidosis. Vitreous fluid was obtained at the beginning of vitrectomy, and centrifuging for cellular removals was not performed. Serum was also collected from PDR patients. IL-1β, IL-4, IL-6, IL-10, IL-17A, IL-17F, IL-21, IL-22, IL-23, IL-25, IL-31, IL-33, IFN-γ, soluble sCD40L, and TNFα in the vitreous and serum samples were measured. Both percent detectable and levels of IL-4, IL-6, IL-17A, IL-21, IL-22, and TNFα in the vitreous were significantly higher than those in the serum in PDR patients. Vitreous levels of these cytokines and IL-31 were significantly higher in PDR than in ERM or MH patients. Vitreous levels of IL-4, IL-17A, IL-22, IL-31, and TNFα in PDR patients were also significantly higher than those of sarcoidosis patients. In PDR patients, vitreous IL-17A level correlated significantly with vitreous levels of IL-22 and IL-31, and especially with IL-4 and TNFα. Although it is unclear whether these cytokines play facilitative roles or inhibitory roles for the progression of PDR, the present study indicated that Th2- and Th17-related immune responses are involved in the pathogenesis of PDR.

## Introduction

Diabetic retinopathy (DR) is one of the most serious complications of diabetes, and is potentially sight-threatening in patients aged 20–75 years [1]. The incidence of DR in adult diabetic patients is higher than 40%, and approximately 5%- 10% of DR cases progress to severe visual impairment [1]. Proliferative diabetic retinopathy (PDR) is characterized by neovascularization followed by fibrovascular changes resulting in vitreous hemorrhage or tractional retinal detachment [2]. Several studies have indicated that inflammatory process is involved in the pathogenesis of DR provoking retinal tissue damage, and that progression of PDR is mediated by various systemic and local factors [2, 3]. Interleukin (IL)-1β [4], IL-6 [5], IL-8 [5], and tumor necrosis factor α (TNFα) [4] are known to be increased in the vitreous fluid of PDR patients, and elevated vitreous levels of C-C motif ligand 2 (CCL2)/monocyte chemoattractant protein-1 (MCP-1), C-X-C motif ligand (CXCL)4/platelet factor (PF)-4, CXCL9/monokine induced by gamma interferon (Mig), and CXCL10/interferon gamma-induced protein (IP)-10 in PDR patients have been reported [6]. Previous studies have demonstrated that macrophages are involved in low-grade inflammation in diabetes, and play pathogenic roles in PDR by producing or expressing the above factors [7, 8]. T cells that are driven by local inflammation act as a conductor in acute and chronic immune responses. Although CD4^+^ and CD8^+^ T cells infiltrate the vitreous of PDR patient and the CD4/CD8 ratio is higher in the vitreous than in the blood [9, 10], the involvement of T cells in progression of PDR has not yet been elucidated.

CD4^+^ T-helper (Th) cells maintain immune homeostasis, and inappropriate differentiation of T cell subsets leads to the development of various inflammatory diseases. Th1 cells that produce mostly interferon (IFN)-γ, IL-2, and TNFβ are implicated in organ-specific autoimmune diseases, while Th2 cells promote humoral immunity by producing IL-4, IL-5, IL-13, and IL-31 [11]. Th17 cells, which are distinct from Th1 or Th2 cells by their ability to produce IL-17 (also called IL-17A), are involved in the development of multiple chronic inflammatory diseases [12]. Th17 cells also preferentially produce IL-17F, IL-21, and IL-22 [13], and have been reported to be associated with the pathogenesis of atherosclerotic diseases [14], adipocyte differentiation, and glucose metabolism [15] by inducing low-grade inflammation. In this study, we measured Th cell-related cytokines in the vitreous fluid of patients with type 2 diabetes-associated PDR, and evaluated T cell subsets related to PDR by comparing vitreous and serum levels of cytokines in PDR and other vitreoretinal diseases.

## Subjects and Methods

### Subjects

The study group consisted of 25 type 2 diabetic patients with PDR who underwent vitrectomy for vitreous hemorrhage and/or tractional retinal detachment between January 1, 2014 and December 31, 2014 in National Defense Medical College. The control group consisted of 27 patients with epiretinal membrane (ERM), 26 patients with idiopathic macular hole (MH), and 26 patients with uveitis associated with sarcoidosis. The exclusion criteria included previous vitrectomy, prior intravitreal therapies, trauma, and infectious endophthalmitis. The clinical characteristics of PDR and control patients are summarized in Table 1. The age (mean ± SD) was 57.0 ± 13.7 years (range 31–83) in PDR group, 61.7 ± 11.4 years (range 39–87) in ERM group, 65.7 ± 6.4 years (range 47–77) in MH group, and 66.3 ± 10.5 years (41–89) in uveitis group. Gender (male/female) ratio was 18/7 in PDR group, 13/14 in ERM group, 9/17 in MH group, and 9/17 in uveitis group. PDR group was significantly younger (*P* < 0.05) and consisted of more males (*P* < 0.05) compared to ERM, MH, or uveitis group. In PDR group, retinal photocoagulation (PC) had been performed in 18 eyes (72%) before referral to our department; total PC in 13 eyes and partial PC in 5 eyes. PC had not been performed in 7 eyes. Vitreous hemorrhage (VH) obscuring fundus findings was observed in 18 of 25 eyes (72%). In 11 eyes (44%) with or suspected of severe tractional retinal detachment caused by active fibrovascular membrane, intravitreal injection of 1.25 mg/0.05 mL bevacizumab (IVB) was performed 2 or 3 days before vitrectomy to minimize intraoperative hemorrhages.

**Table 1:** Characteristics of proliferative diabetic retinopathy (PDR) and control [idiopathic epiretinal membrane (ERM), idiopathic macular hole (MH), and uveitis associated with sarcoidosis] patients.

	PDR (n = 25)	ERM (n = 27)	MH (n = 26)	Uveitis (n = 26)
**Age (year)**	57.0 ± 13.7^*^ (31–83^†^)	61.7 ± 11.4 (39–87) †	65.7 ± 6.4 (47–77)	66.3 ± 10.5 (41–89)
**Gender (M/F)**	18/7	13/14	9/17	9/17
**Photocoagulation**	18 (72.0) ^**‡**^			
**Pan**	13			
**Partial**	5			
**Vitreous hemorrhage**	18 (72.0)			
**IVB** ^§^	11 (44.0)			

^*^ Mean ± standard deviation.

^†^ Range.

^**‡**^ Numbers (%).

^§^ IVB = intravitreal injection of bevacizumab.

### Ethics statement

The study was approved by the Institutional Review Board of National Defense Medical College. Eligible participants were informed about the purpose and experimental procedure of the study, and signed a copy of the Institutional Review Board approved consent form prior to participation.

### Sample collection

Approximately 0.2 to 0.5 mL of undiluted vitreous fluid was obtained using a 25G vitreous cutter inserted into the mid-vitreous cavity at the beginning of surgery before active infusion. In patients performed surgery for both eyes, we used the vitreous samples collected from the first operated eye. The vitreous samples were not centrifuged for cellular removals and were transferred into sterile tubes and stored at -70°C until analysis. No complication associated with vitreous sampling was observed. Peripheral blood was collected before surgery from PDR patients from whom vitreous samples were also collected. Serum was separated by centrifugation at +4°C. The serum samples were also frozen at –70°C until use.

### Cytokine measurements

Cytokines in the vitreous and serum samples were measured by the Bio-Plex Pro™ Human Th17 Cytokine Assays^®^ (Bio-Rad Laboratories, Inc.). In brief, the samples were diluted 4-fold with the diluting solution, and centrifuged at 10,000 × g for 5 minutes. Fifty *μ*L of the supernatant was used for the cytokine assay in accordance with the manufacturer's instructions. The following cytokines were measured: IL-1β, IL-4, IL-6, IL-10, IL-17A, IL-17F, IL-21, IL-22, IL-23, IL-25, IL-31, IL-33, IFNγ, soluble CD40 ligand (sCD40L), and TNFα. The lower limits of detection according to our standard curves were 0.27–0.28 pg/ml for IL-1β, 1.3–4.3 pg/ml for IL-4, 0.9–2.6 pg/ml for IL-6, 2.0–3.7 pg/ml for IL-10, 1.5–2.0 pg/ml for IL-17A, 1.8–8.0 pg/ml for IL-17F, 5.0–20.5 pg/ml for IL-21, 4.6–5.0 pg/ml for IL-22, 7.0–26.1 pg/ml for IL-23, 1.2–1.4 pg/ml for IL-25, 2.8–4.8 pg/ml for IL-31, 3.3–7.8 pg/ml for IL-33, 3.2–3.9 pg/ml for IFNγ, 3.2–7.4 pg/ml for sCD40L, and 0.3–2.4 pg/ml for TNFα.

### Statistical analysis

Cytokines in vitreous fluid and serum are presented in percent of samples with detectable cytokine (percent detectable, %) and in cytokine level (mean ± standard deviation, pg/ml) for each group. Cytokines with values below limits of detection were graded as negative and the levels were assigned a numerical value of 0 pg/ml for statistical analysis. Percent detectable were compared by Pearson's chi-squared test. Since the levels of cytokines were not normally distributed, the statistical comparisons of cytokine levels were performed using nonparametric test (Wilcoxon signed-rank test for two groups, and Steel-Dwass multiple comparison test for three groups). Pearson’s correlation coefficient test was used to examine the correlation between vitreous levels of cytokines. A *P* value of 0.05 was considered significant.

## Results

### Comparison of vitreous and serum cytokine levels

Percent detectable and levels of cytokines in serum and vitreous samples collected from 25 PDR patients who underwent vitrectomy are shown in Table 2. Cytokines with percent detectable higher than 20% either in the serum or in the vitreous were IL-4, IL-6, IL-10, IL-17A, IL-21, IL-22, IL-31, sCD40L, and TNFα. Percent detectable of IL-31 and sCD40L were higher in the serum than in the vitreous, and the difference was significant for sCD40L. However, percent detectable of the other cytokines; IL-4, IL-6, IL-10, IL-17A, IL-21, IL-22, and TNFα, were significantly higher in the vitreous than in the serum. Especially, IL-4, IL-10, IL-17A, IL-21, and IL-22 were only detected in the vitreous. Similar to percent detectable, mean levels of IL4, IL-6, IL-10, IL-17A, IL-21, IL-22, and TNFα were significantly higher in the vitreous than in the serum. Contrary to the percent detectable, mean level of IL-31 was significantly higher in the vitreous than in the serum.

**Table 2:** Cytokines detected in vitreous fluid and serum samples collected from patients with proliferative diabetic retinopathy.

	Vitreous samples (n = 25)		Serum samples (n = 25)	
	Detectable samples (%)	Level (pg/ml) mean ± SD	Detectable samples (%)	Level (pg/ml) mean ± SD
**IL-1β**	2 (8)	0.1 ± 0.4	2 (8)	0.5 ± 2.1
**IL-4**	16 (64) ^*^	86.8 ± 111.9^†^	0 (0)	0
**IL-6**	25 (100)^*^	658.5 ± 402.1^†^	3 (12)	2.82 ± 10.2
**IL-10**	5 (20)^*^	2.9 ± 7.5^†^	0 (0)	0
**IL-17A**	15 (60)^*^	274.3 ± 327.5^†^	0 (0)	0
**IL-17F**	2 (8)	3.0 ± 10.6	2 (8)	10.2 ± 50.0
**IL-21**	8 (32)^*^	448.8 ± 965.7^†^	0 (0)	0
**IL-22**	17 (68)^*^	51.3 ± 84.3^†^	0 (0)	0
**IL-23**	0 (0)	0	0 (0)	0
**IL-25**	0 (0)	0	3 (12)	1.4 ± 4.8
**IL-31**	16 (64)	195.6 ± 263.5^†^	19 (76)	60.3 ± 53.4
**IL-33**	2 (8)	17.1 ± 60.9	0 (0)	0
**IFN-γ**	2 (8)	4.1 ± 16.3	2 (8)	26.3 ± 92.4
**sCD40L**	1 (4)	3.3 ± 16.4	19 (76.0)^*^	935.3 ± 901.0^†^
**TNFα**	25 (100) ^*^	55.3 ± 56.3^†^	2 (8.0)	0.11 ± 0.4

**Detectable samples (%)**: Number (%) of samples with detectable cytokine.

^*^ *P* < 0.05 vitreous vs. serum, by Pearson's chi-squared test.

† *P* < 0.05 vitreous vs. serum, by Wilcoxon signed-rank test.

### Comparison of vitreous cytokines in PDR with and without vitreous hemorrhage

Since we assayed whole vitreous samples without removing the cells, PMBC from blood in samples from patients with vitreous hemorrhage may interfere with vitreous cytokine levels. Therefore, we compared the vitreous levels of cytokines between PDR eyes with and without VH. Percents detectable and levels of vitreous cytokines in PDR with or without VH are shown in Table 3. There were no significant differences between PDR patients with and those without VH both in percents detectable and mean vitreous levels of all the cytokines tested.

**Table 3:** Comparison of vitreous cytokine levels between proliferative diabetic retinopathy (PDR) with and without vitreous hemorrhages (VH).

	PDR with VH (n = 18)		PCR without VH (n = 7)	
	Detectable samples (%)	Level (pg/ml) mean ± SD	Detectable samples (%)	Level (pg/ml) mean ± SD
**IL-1β**	2 (11.1)	0.1 ± 0.5	0 (0)	0
**IL-4**	12 (66.7)	92.1 ± 120.2	4 (57.1)	72.9 ± 93.9
**IL-6**	18 (100)	676.0 ± 375.6	7 (100)	613.3 ± 493.7
**IL-10**	3 (16.7)	3.5 ± 8.7	2 (28.6)	1.5 ± 2.5
**IL-17A**	12 (66.7)	315.3 ± 325.1	3 (42.9)	168.9 ± 244.1
**IL-17F**	1 (5.6)	2.5 ± 10.6	1 (14.3)	4.3 ± 10.3
**IL-21**	5 (27.8)	324.5 ± 834.0	3 (42.9)	768.4 ± 1262.6
**IL-22**	13 (72.2)	40.0 ± 51.0	4 (57.1)	80.5 ± 140.1
**IL-23**	0 (0)	0	0 (0)	0
**IL-25**	0 (0)	0	0 (0)	0
**IL-31**	12 (66.7)	202.4 ± 282.4	4 (57.1)	178.0 ± 226.5
**IL-33**	1 (5.6)	14.7 ± 62.4	1 (14.3)	23.1 ± 61.1
**IFN-γ**	1 (5.6)	4.4 ± 18.7	1 (14.3)	3.3 ± 8.7
**sCD40L**	1 (5.6)	4.6 ± 19.3	0 (0)	0
**TNFα**	18 (100)	61.5 ± 55.1	7 (100)	39.5 ± 60.7

**Detectable samples (%)**: Number (%) of samples with detectable cytokine.

### Comparison of vitreous cytokines in PDR and other idiopathic retinal diseases

**Fig 1:**
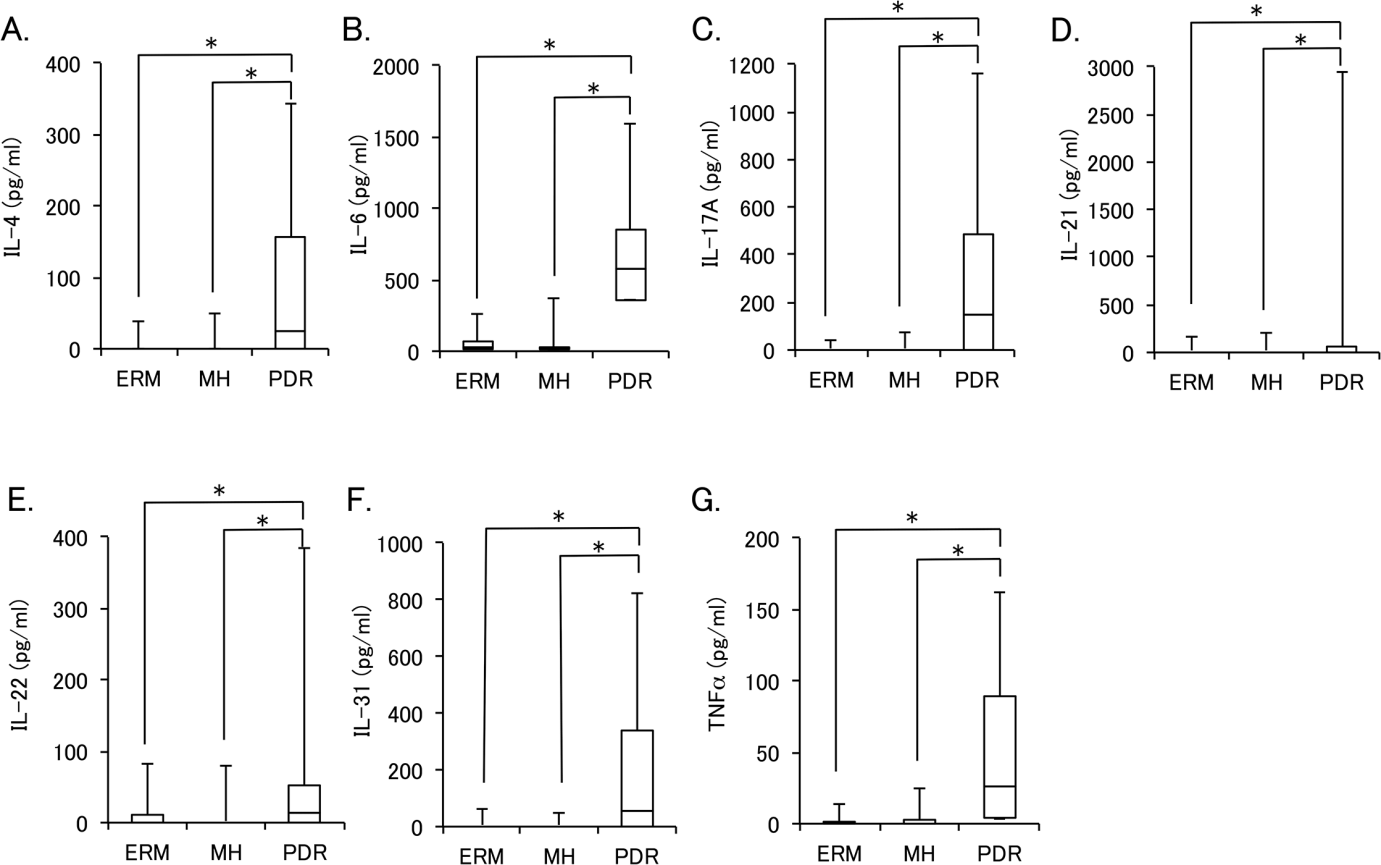
Vitreous levels of cytokines in proliferative diabetic retinopathy (PDR), epiretinal membrane (ERM), and macular hole (MH). The mean vitreous levels of IL-4 (A), IL-6 (B), IL-17A (C), IL-21 (D), IL-22 (E), IL-31 (F), and TNFα (G) in PDR, ERM and MH group are shown as box plots with median and quartile values. *Significant difference analyzed by Steel-Dwass multiple comparison test (P < 0.05).

Percent detectable and levels of vitreous cytokines in PDR were compared with those in other retinal diseases (Table 4). When comparing PDR with idiopathic retinal diseases (ERM and MH), while percent detectable of IL-1β, IL-17F, IL-23, IL-25, IL-33, IFN-γ, and sCD40L were less than 20% and that of IL-6 was higher than 90% in all three groups, those of IL-4, IL-10, IL-17A, IL-21, IL-22, IL-31, and TNFα were significantly higher in PDR group than in ERM or MH group. Fig 1 shows the box plots of vitreous levels of IL-4, IL-6, IL-17A, IL-21, IL-22, IL-31, and TNFα in PDR, ERM, and MH groups. The vitreous levels of IL-4, IL-6, IL-17A, IL-21, IL-22, IL-31, and TNFα were significantly higher in PDR group than in ERM or MH group.

**Table 4:** Comparison of vitreous cytokine levels between proliferative diabetic retinopathy (PDR), idiopathic epiretinal membrane (ERM), idiopathic macular hole (MH), and uveitis associated with sarcoidosis.

	PDR (n = 25)		ERM (n = 27)		MH (n = 26)		Uveitis (n = 26)	
	Detectable samples (%)	Level (pg/ml) mean ± SD	Detectable samples (%)	Level (pg/ml) mean ± SD	Detectable samples (%)	Level (pg/ml) mean ± SD	Detectable samples (%)	Level (pg/ml) mean ± SD
**IL-1β**	2 (8)	0.1 ± 0.4	0 (0)	0	1 (3.8)	0.1 ± 0.7	3 (11.5)	0.2 ± 0.7
**IL-4**	16 (64) ^*^ ^†^	86.8 ± 111.9^‡^ ^§^	3 (11.1)	3.1 ± 9.0	2 (7.7)	3.6 ± 12.0	7 (26.9)	10.2 ± 24.6
**IL-6**	25 (100)	658.5 ± 402.1^‡^	27 (100)	45.7 ± 54.9	25 (96.2)	39.6 ± 71.8	26 (100)	2707.7 ± 4973.5
**IL-10**	5 (20)^*^	2.9 ± 7.5^‡^	0 (0)	0	0 (0)	0	10 (38.5)	6.8 ± 12.0
**IL-17A**	15 (60)^*^	274.3 ± 327.5^‡^ ^§^	4 (14.8)	2.6 ± 8.5	4 (11.5)	4.2 ± 14.7	9 (34.6)	20.9 ± 54.4
**IL-17F**	2 (8)	3.0 ± 10.6	0 (0)	0	0 (0)	0	5 (19.2)	7.3 ± 18.9
**IL-21**	8 (32)^*^	448.8 ± 965.7^‡^	1 (3.7)	6.2 ± 32.3	1 (3.8)	7.8 ± 39.8	4 (15.4)	14.6 ± 44.6
**IL-22**	17 (68) ^*^ ^†^	51.3 ± 84.3^‡^ ^§^	11 (40.7)	8.0 ± 16.1	5 (19.2)	5.3 ± 16.1	5 (19.2)	6.0 ± 16.3
**IL-23**	0 (0)	0	0 (0)	0	0 (0)	0	0 (0)	0
**IL-25**	0 (0)	0	0 (0)	0	0 (0)	0	2 (7.7)	0.2 ± 0.7
**IL-31**	16 (64) ^*^	195.6 ± 263.5^‡^ ^§^	2 (7.4)	2.8 ± 12.3	1 (3.8)	1.9 ± 9.6	14 (53.9)	16.7 ±20.3
**IL-33**	2 (8)	17.1 ± 60.9	0 (0)	0	0 (0)	0	0 (0)	0
**IFN-γ**	2 (8)	4.1 ± 16.3	1 (3.7)	0.3 ± 1.5	1 (3.8)	1.6 ± 8.0	13 (50)^†^	31.9 ± 52.3^§^
**sCD40L**	1 (4)	3.3 ± 16.4	1 (3.7)	3.8 ± 19.8	1 (3.8)	1.2 ± 6.0	8 (30.8)^†^	18.2 ± 35.8^§^
**TNFα**	25 (100)^*^	55.3 ± 56.3^‡^ ^§^	9 (33.3)	2.1 ± 4.0	10 (38.5)	2.6 ± 5.6	23 (88.5)	10.6 ± 11.8

**Detectable samples (%)**: Number (%) of samples with detectable cytokine.e

^*^ *P* < 0.05 between PDR, MH, and ERM, by Pearson's chi-squared test.

^†^ *P* < 0.05 between PDR and uveitis, by Pearson's chi-squared test.

^‡^ *P* < 0.05 between PDR, MH, and ERM, by Steel-Dwass multiple comparison test.

^§^ *P* < 0.05 between PDR and uveitis, by Wilcoxon signed-rank test.

### Comparison of vitreous cytokines in PDR and inflammatory disease

**Fig 2:**
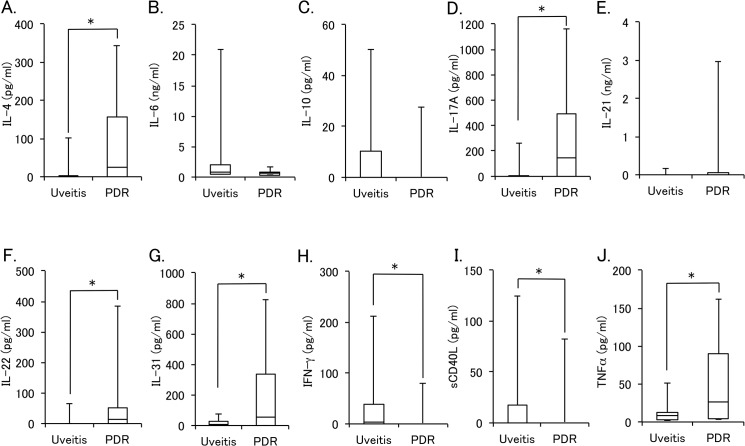
Vitreous levels of cytokines in proliferative diabetic retinopathy (PDR) and uveitis associated sarcoidosis. The mean vitreous levels of IL-4 (A), IL-6 (B), IL-10 (C), IL-17A (D), IL-21 (E), IL-22 (F), IL-31 (G), IFN-γ (H), sCD40L (I), and TNF-α (J) in PDR and uveitis group are shown as box plots with median and quartile values. *Significant difference analyzed by Steel-Dwass multiple comparison test (P < 0.05).

Percent detectable and levels of vitreous cytokines in PDR were also compared with those in uveitis associated with sarcoidosis. As shown in Table 4, percent detectable of IL-4 and IL-22 were significantly higher in PDR group than in uveitis group, although those of IFN-γ and sCD40L were significantly higher in uveitis group compared with PDR group. Fig 2 shows the box plots of vitreous levels of IL-4, IL-6, IL-10, IL-17A, IL-21, IL-22, IL-31, IFN-γ, sCD40L, and TNFα in PDR and uveitis groups. Similar to the result of percent detectable, the vitreous levels of IFN-γ and sCD40L were significantly higher in uveitis group than in PDR group, whereas those of IL-4, IL-17A, IL-22, IL-31, and TNFα were significantly higher in PDR group compared with uveitis group.

### Correlation of vitreous level of IL-17A with those of other cytokines elevated in PDR patients

**Fig 3:**
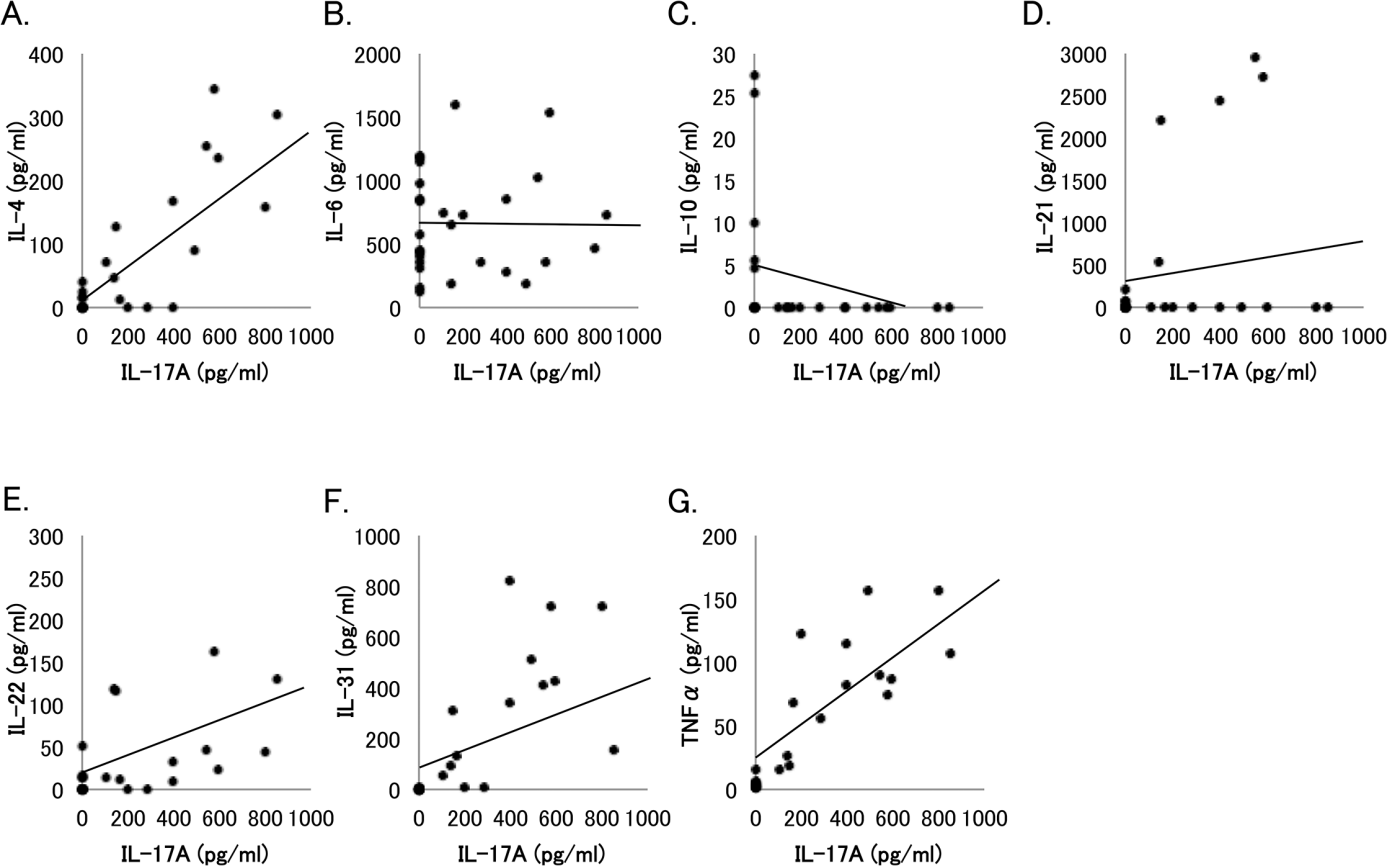
Association of vitreous level of IL-17A with vitreous levels of other cytokines in patients with proliferative diabetic retinopathy (PDR). Correlation of IL-17A with (A) IL-4 (y = 9.8 + 0.28x, R_2_ = 0.67, *P* < 0.0001), (B) IL-6 (y = 660.0–0.006x, R_2_ = 0.0002, P = 0.9824), (C) IL-10 (y = 5.06–0.008x, R_2_ = 0.1171, P = 0.0941), (D) IL-21 (y = 291.1 + 0.57x, R_2_ = 0.0380, P = 0.3505), (E) IL-22 (y = 22.1 + 0.11x, R_2_ = 0.1708, *P* = 0.04), (F) IL-31 (y = 61.8 + 0.49x, R_2_ = 0.3671, *P* = 0.0013), or (G) TNFα (y = 14.4 + 0.15x, R_2_ = 0.7539, *P* < 0.0001) in PDR patients was analyzed by Pearson’s correlation coefficient test.

Fig 3 presents the correlation of vitreous level of IL-17A with those of other cytokines that were significantly elevated in PDR group compared to ERM, MH, or uveitis group. A significant correlation was observed between IL-17A and IL-4 (y = 9.8 + 0.28x, R_2_ = 0.67, *P* < 0.0001), IL-22 (y = 22.1 + 0.11x, R_2_ = 0.1708, *P* < 0.05), IL-31 (y = 61.8 + 0.49x, R_2_ = 0.3671, *P* < 0.005), or TNFα (y = 14.4 + 0.15x, R_2_ = 0.7539, *P* < 0.0001). No correlation was observed between IL-17A and IL-6, IL-10, or IL-21.

## Discussion

In the present study, we attempted to investigate the vitreous cytokine profile specific to PDR. IL-4, IL-6, IL-17A, IL-21, IL-22 and TNFα levels were significantly higher in the vitreous than in the serum of PDR patients. Vitreous levels of these cytokines plus IL-31 were significantly higher in PDR than in ERM, MH or uveitis. In PDR patients, vitreous IL-17A level correlated significantly with vitreous levels of IL-4, IL-22, IL-31, and TNFα. A major technical issue of this study was that whole vitreous samples were assayed without removing the cells. It is possible that cytokines released by PBMC present in vitreous samples of patients with VH may affect the results. However, leukocytes are only about 0.1% of cells in a hemorrhage. In fact, we found no significant differences in percents detectable and levels of vitreous cytokines between PDR patients with VH and those without VH, suggesting that the presence of blood cells in vitreous samples had no significant effect on the overall results. In addition, since the amounts of intracellular cytokines are very few, those can not be analyzed by quantitative methods used for extracellular cytokine measurement [16], Therefore, it is unlikely that intracellular cytokines of vitreous cells including PBMC contributed to the vitreous cytokine levels.

IL-6 and TNFα are well known proinflammatory cytokines secreted by macrophages and neutrophils. These two cytokines have been shown to increase in the vitreous of PDR patients [5, 17–20], consistent with the result in the present study. In this study, although vitreous level of IL-6 was significantly higher in PDR group than in ERM and MH group, IL-6 was detected in 107 of 108 patients tested. Thus, IL-6 expression in vitreous does not seem to be a specific change in PDR.

IL-17 is implicated in amplifying the immune response by triggering the production of proinflammatory cytokines such as IL-6, TNFα, and IL-1β, facilitating a link between T cell activation and inflammation [21]. In addition, Th17 cells are known to secrete IL-6 and TNFα potentially [12, 22, 23]. In the present study, since vitreous level of IL-17A did not correlate with that of IL-6 but did correlate with TNFα, it is possible that TNFα produced by Th17 cells contributes at least partially to the TNFα found in the vitreous of PDR patients.

Some studies have indicated that vitreous level of IL-10 in DR patients is significantly higher than that in patients with ERM or MH [19, 24]. This finding is compatible with the present study in which IL-10 was detected in PDR patients was 24.1% (7 of 29 eyes) and IL-10 was not detected in the vitreous of patients with ERM or MH. On the contrary, other studies reported that IL-10 was not detected or elevated in vitreous fluid of DR patients [18, 25]. These discrepant findings may be caused by different methods used and variable sensitivities of different assay systems. Besides, because most diabetic patients have comorbidities such as hypertension and arterial sclerosis, the repertoire, differentiation, and activities of immune cells involved in pathomechanisms would vary greatly within the population of DR patients.

IL-31 is a cytokine structurally associated with IL-6, and is preferentially produced by Th2 cells [26]. The function of IL-31 is not fully understood, but this cytokine has been associated with atopic dermatitis [27], Crohn disease [28], and allergic asthma and rhinitis [29]. Although the involvement of IL-31 in diabetes is not known, IL-31 was detected in both the serum and vitreous samples of PDR patients (76% and 58.6%, respectively), and the percent detectable and level in the vitreous fluid of PDR patients were significantly higher than those in patients with ERM or MH, suggesting a possible role of IL-31 in the pathogenesis of diabetes.

In most cases of uveitis associated with sarcoidosis, vitrectomy is performed for complications refractory to medical treatment, such as vitreous opacity and/or ERM, during remission of uveitis. However, percent detectable of IL-10, IL-31, IFN-γ, sCD40L, and TNFα, and mean vitreous levels of IL-6, IL-10, IL-31, IFN-γ, sCD40L, and TNFα were significantly higher in eyes with uveitis than in those with ERM or MH (data not shown). These results indicate underlying inflammation in eyes with sarcoidosis even during the remission phase. In addition, percent detectable and levels of IFN-γ and sCD40L in the vitreous of eyes with sarcoidosis were also significantly greater than those in eyes with PDR, suggesting that Th1-related immune responses are more critical in uveitis associated with sarcoidosis than Th2 responses.

Since IVB was performed 2 or 3 days before vitrectomy in 13 eyes of PDR patients, we compared percent detectable and mean levels of cytokines in vitreous fluid between PDR patients who received IVB and those who did not. However, in this study population, no cytokine was significantly higher in PDR patients who received IVB before vitrectomy compared with those who did not (data not shown). However, the effects of IVB together with other factors such as age, fasting blood glucose, HbA1c, and insulin on vitreous cytokines in eyes with PDR are being evaluated in a prospective randomized study with a larger number of PDR patients.

## Conclusions

The present study demonstrated that: 1) both percent detectable and levels of IL4, IL-6, IL-17A, IL-21, IL-22, and TNFα in the vitreous of PDR patients were significantly higher than those in the serum; 2) vitreous levels of the above cytokines and IL-31 were significantly higher in PDR than in other non-inflammatory vitreoretinal diseases; 3) vitreous levels of IL-4, IL-17A, IL-22, IL-31, and TNFα were also significantly higher in PDR than in uveitis associated with sarcoidosis; and 4) vitreous level of IL-17A in PDR patients correlated significantly with those of IL-4, IL-22, IL-31, and TNFα. Although it is unclear whether these cytokines play facilitative roles or inhibitory roles for the progression of PDR, the present study indicated that Th2- and Th17-related immune responses are involved in the pathogenesis of PDR.
